# Engineered butyrate-producing yeasts mitigate Alzheimer-associated phenotypes

**DOI:** 10.1038/s41392-025-02474-7

**Published:** 2025-11-17

**Authors:** Biao Zhang, Zhiyu Sun, Weihong Song, He Huang, Yili Wu

**Affiliations:** 1 School of Synthetic Biology and Biomanufacturing, State Key Laboratory of Synthetic Biology, Tianjin Key Laboratory of Biological and Pharmaceutical Engineering, Tianjin University, Tianjin, China; 2 Wenzhou Key Laboratory of Basic and Translational Research for Mental disorders, Key Laboratory of Alzheimer’s Disease of Zhejiang Province, Zhejiang Provincial Clinical Research Center for Mental Health, School of Mental Health and The Affiliated Kangning Hospital, Institute of Aging, Oujiang Laboratory (Zhejiang Lab for Regenerative Medicine, Vision and Brain Health), Wenzhou Medical University, Wenzhou, Zhejiang China

**Keywords:** Translational research, Diseases of the nervous system

**Dear Editor**,

Alzheimer’s disease (AD) is the most prevalent neurodegenerative disease without effective treatments.^1^ Extracellular amyloid plaques, neurofibrillary tangles, neuroinflammation, and neuronal loss are the characteristics of AD neuropathology. Amyloid β protein (Aβ), the major component of amyloid plaques, is produced through the sequential cleavage of the amyloid precursor protein (APP) by β-secretase BACE1 and γ-secretase.^1^ Dysregulation of APP, BACE1, and γ-secretase increases Aβ generation while dysfunction of microglia reduces Aβ clearance, contributing to Aβ deposition.^2^ The increase in Aβ deposition simultaneously activates microglia, promoting the secretion of a large number of pro-inflammatory cytokines. Dysregulation of gut microbiota and their metabolites plays a pivotal role in AD pathogenesis.^3^ Among the metabolites, butyrate has beneficial effects on AD. Increasing the bioavailability of butyrate is critical for its clinical application.^4^ To achieve consistent supplementation of butyrate, we have developed a strain of engineered butyrate-producing *Saccharomyces cerevisiae* (J17), a yeast probiotic,^5^ which may mitigate AD phenotypes by the synergistic effect of butyrate supplementation and probiotic function of the chassis.

We first investigated whether butyrate is deficient in the blood and brain of APP23/PS45 mice, a strain of AD model mice, and whether J17 has beneficial effects on AD. In APP23/PS45 mice, the butyrate levels were significantly decreased to 53.99 ± 6.90% (blood, *P* < 0.05) and 28.92 ± 2.92% (brain, *P* < 0.01) of those in the wild-type (WT) mice, respectively, which is consistent with that in AD patients and in the other two AD model mice, APP/PS1 mice and 3xTg mice. J17 administration rescued butyrate deficiency in the blood and brain of the AD mice, while the chassis strain BY4741 did not (Fig. 1a). Importantly, J17 rescued cognitive impairment in the AD mice. In the Morris water maze (MWM) test, no difference of escape latency was observed on the 1st day of visible platform trial among the four groups. From the 2nd day to the 5th day, the hidden platform trials were performed. The escape latency in the J17-treated AD mice was significantly reduced compared with that in the AD mice (e.g., 27.82 ± 5.86 seconds vs. 50.20 ± 6.64 seconds on the 5th day, *P* < 0.05), while it was comparable to that in the WT mice (30.01 ± 4.70 seconds) (Fig. 1b). In the probe trial of the last day of MWM testing, the AD mice swam significantly shorter distances in the target quadrant compared with the WT mice (Fig. 1b). J17-treated AD mice swam significantly longer distances in the target quadrant than the AD mice did, respectively (Fig. 1b). Notably, a trend of the increase of distance was observed in BY4741-treated AD mice, although the difference was not statistically significant. These results demonstrate that J17 rescues the learning and memory deficits of the AD mice, which may be attributed to the synergistic effect of butyrate supplementation and the probiotic function of the chassis.

**Fig. 1:**
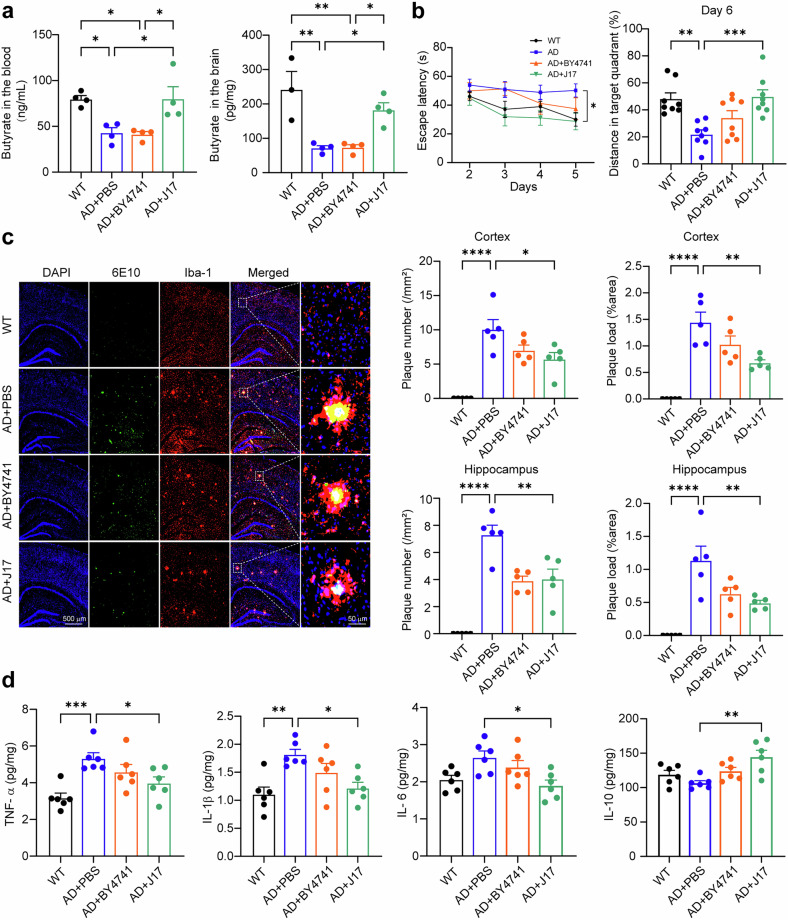
Engineered butyrate-producing yeasts (J17) rescue butyrate deficiency and mitigate AD-associated phenotypes in APP23/PS45 mice. **a** The levels of butyrate in the blood and brain of the mice. *n* = 3-4 per group. **b** The escape latency of the 2nd to 5th day training phase in the MWM test, and the swimming distance in the last day probe trial of the MWM probe test. *n* = 8. **c** Representative images of immunofluorescence staining for nuclei (blue), Aβ plaques (green), and Iba-1 (red) with DAPI, anti-Aβ (6E10) and anti-Iba-1 antibodies, respectively. Scale bar: 500 μm or 50 μm. The number and size of Aβ plaques in the cortex and hippocampus. *n* = 5. **d** The levels of TNF-α, IL-1β, IL-6, and IL-10 in the cortex of mice were detected by ELISA. *n* = 6. Statistical analysis was performed using one-way ANOVA followed by Tukey’s multiple comparison test. Data represent the means ± SEM. *P < 0.05, **P < 0.01, ***P < 0.001, ****P < 0.0001

To examine the effect of J17 on Aβ deposition, immunofluorescence staining with Aβ antibody (6E10) was performed. J17 application significantly reduced the number and size of Aβ plaques to 5.70 ± 2.20 /mm^2^, 0.68 ± 0.13% area and 4.03 ± 1.64 /mm^2^, 0.49 ± 0.10% area (*P* < 0.05) in the cortex and hippocampus of AD mice, respectively, compared with 10.06 ± 3.21 /mm^2^, 1.44 ± 0.43% area and 7.31 ± 1.57 /mm^2^, 1.14 ± 0.48% area in the cortex and hippocampus of AD mice treated with PBS, respectively. In contrast, a trend of the decrease in the number and size of Aβ plaques was observed in BY4741-treated AD mice, although the difference was not statistically significant (Fig. 1c). This indicates that the effect of J17 on Aβ deposition may be attributed to the synergistic effect of butyrate supplementation and probiotic function of the chassis. To explore underlying mechanisms, we further investigated APP processing. Compared with AD mice, the levels of APP, BACE1, and CTF_β_ were significantly decreased in the cortex of J17-treated AD mice, respectively, while Aβ42 was significantly decreased too (data not shown). It indicates that J17 reduces Aβ plaque formation and Aβ42 generation by inhibiting BACE1-mediated β-cleavage of APP.

In addition to Aβ generation, reduced Aβ clearance also contributes to increased Aβ and its deposition, which is associated with abnormal microglia activation. We found that the J17 mitigated microglia activation in AD mice, indicated by the immunofluorescence staining of Iba-1 (Fig. 1c). Consistently, J17 decreased the levels of proinflammatory cytokine TNF-α, IL-1β, and IL-6 in the cortex to 74.85 ± 15.56%, 66.91 ± 11.74% and 71.45 ± 14.29% (*P* < 0.05), respectively. However, the level of anti-inflammatory cytokine IL-10 was increased to 135.63 ± 6.84%, *P* < 0.05 (Fig. 1d). Moreover, consistent alterations were observed in the hippocampus (data not shown). It indicates that J17 mitigated microglia overactivation and neuroinflammation in the AD mice, which may contribute to the reduced Aβ levels and Aβ deposition by facilitating microglia-mediated Aβ clearance.

Furthermore, J17 reshaped the gut microbiota and reduced inflammation- and neurotoxicity-related metabolites in the AD mice (data not shown). It indicates that J17 is beneficial for healthy gut environment maintenance in the AD mice, which may contribute to mitigating AD-associated phenotypes.

In this study, we demonstrated that J17 rescues butyrate deficiency and cognitive deficits, and mitigates Aβ deposition, microglia overactivation, and neuroinflammation in APP23/PS45 mice. Notably, BY4741 shows a trend for the amelioration of AD-associated phenotypes without the alteration of butyrate, however, the difference was not statistically significant. It highly suggests the beneficial effect of J17 on AD may be attributed to the synergistic effect of butyrate supplementation and the probiotic function of BY4741. Compared with fecal microbiota transplantation, the administration of J17 has advantage of avoiding the potential risk of unknown or conditional pathogens for AD treatment. However, the dosage-effect and long-term effect of J17 should be investigated in future studies.

## Supplementary information

Supplementary Materials for Engineered butyrate-producing yeasts mitigate Alzheimer-associated phenotypes

## Data Availability

16s rRNA sequencing data have been deposited in the BioProject database (http://www.ncbi.nlm.nih.gov/bioproject/1175887), BioProject ID: 1175887. Non-targeted metabolomics data have been deposited in the ScienceDB database (https://www.scidb.cn/anonymous/YkVGZkVm). The other data that support the findings of this study are available from the corresponding author upon request.
