# Supplementary material for: Engineered butyrate-producing yeasts mitigate Alzheimer-associated phenotypes
Source: Signal Transduct Target Ther. 2025 Nov 17;10:369. doi: 10.1038/s41392-025-02474-7 (PMC12620515; doi:10.1038/s41392-025-02474-7)
Supplement: Supplementary file 1 — Supplementary Materials for Engineered butyrate-producing yeasts mitigate Alzheimer-associated phenotypes [file 41392_2025_2474_MOESM1_ESM.docx]

Supplementary Materials for

Engineered butyrate-producing yeasts mitigate Alzheimer-associated phenotypes

Biao Zhang^1,2†^, Zhiyu Sun^2†^, Weihong Song^2^, He Huang^1 *^, Yili Wu^2 *^

Correspondence to: [wuyili@wmu.edu.cn](mailto:wuyili@wmu.edu.cn)

**This PDF file includes:**

Materials and Methods

References

Materials and Methods

**Animal and Treatment**

APP23 mice overexpressing the Swedish APP751 (KM→NL) mutant transgene driven by the murine Thy1.2 promoter were originally generated at Novartis Pharma as previously described^1,2^. PS45 mice overexpressing the human G384A-mutated PS1 under the control of the murine Thy1 promoter^3^. APP23/PS45 double transgenic mice were generated by crossing APP23 mice with PS45 mice^4-6^. The double-transgenic mice develop detectable neuritic plaques in the cortex and hippocampus as early as 1 month of age and exhibit cognitive deficits as early as 2.5 month of age^3-6^.

7-week-old APP23/PS45 double-transgenic mice (AD mice) and their wild-type (WT) littermates were applied for one-week adaptive feeding. At 8 weeks old, APP23/PS45 mice were randomly treated with PBS (AD), chassis strain BY4741 (AD+BY4741) and engineered butyrate-producing *S. cerevisiae* J17 (AD+J17) via gavage, respectively, while the WT mice were administrated with PBS (WT group). The dosages of BY4741 and J17 were 2×10^8^ cfu/time/day. After 4-week treatment, the behavioral tests were performed.

**Morris water maze test**

In brief, the test was performed in a 1.5-meter-diameter pool with a 10-cm-diameter platform placed in the SW quadrant of the pool. The procedure consisted of 1 day of visible platform tests and 4 days of hidden platform tests, plus a probe trial after the last hidden platform test. The mice conducted four tests each day from four different starting points. In the visible platform test performed on the 1^st^ day, the mice were tested for 4 continuous trials. From the 2^nd^ day to the 5^th^ day, titanium dioxide is used to make the water cloudy, concealing the submerged platform. Each mice has 60 seconds to search for the platform. If the mice fails to locate the platform, the experiment will guide it to the platform and allow the mice to rest there for 15 seconds. After the last trial on the 5^th^ day, the platform is removed from the pool. Twenty-four hours later, on the 6^th^ day, the mice are given a 60 seconds swimming probe trial test. The time spent and distance traveled in the quadrant where the platform was located, are recorded. The ANY-maze video tracking system is used for data collection.

**Detection of butyrate levels**

The mice blood and lysates of whole half brain were applied for the butyrate assessment. The Thermo Trace 1300 meteorological system (Thermo Fisher Scientific, USA) was utilized, equipped with an Agilent HP-INNOVAX capillary column (30 m × 0.25 mm ID × 0.25 μm) for chromatographic separation. Split injection was employed, with an injection volume of 1 μL and a split ratio of 10:1. The injection port temperature was set at 250 ℃, while the ion source temperature was maintained at 300 ℃. The transmission line temperature was adjusted to 250 ℃. The temperature program for the oven began at 90 ℃, followed by an increase to 120 ℃ at a rate of 10 ℃/min. Subsequently, the temperature was elevated at a rate of 5 ℃/min to reach 150 ℃. Finally, the temperature was raised to 250 ℃ at a rate of 25 ℃/min and held for 2 minutes. Helium served as the carrier gas, with a flow rate of 1.0 mL/min.

**Immunofluorescence**

Immunofluorescence analysis was performed as previously described^7^. The mice brains were fixed, dehydrated, embedded and frozen. They were then sectioned into 20 μm slices. After washing and blocking, the slices were incubated with the corresponding primary antibodies overnight. The next day, the primary antibodies on the surface of the slices was discarded. The slices were then immersed in a staining tank filled with PBS and gently shaken on a shaker for 3 washes, each lasting 5 minutes, to remove the unbound primary antibodies. The fluorescently labeled secondary antibody was prepared. Under light-protected conditions, the secondary antibody solution was dropped onto the slices and incubated at room temperature in the dark for 1 hour. Following this, the slices were washed and imaged. Image analysis was performed using Image J analysis software (NIH). The following antibodies were applied: anti-amyloid beta (6E10) (BioLegend, 803001, 1:400) and anti-Iba-1 (Cell Signaling Technology, 17198S, 1:200). DAPI (Sigma-Aldrich, D95542, 1 μg/mL).

**Enzyme linked immunosorbent assay**

The cortical/hippocampal tissues were prepared according to the manufacturer’s instructions prior to carrying out the ELISA protocol. In brief, the cortical/hippocampal tissues were homogenized in ice-cold PBS containing protease inhibitors. The homogenate was centrifuged at 12,000×g, 4 °C for 10 minutes and the supernatant was collected for ELISA. The following ELISA Kits were used: TNF-α (MULTI SCIENCES, EK282HS), IL-6 (MULTI SCIENCES, EK206HS), IL-1β (MULTI SCIENCES, EK201BHS) and IL-10 (MULTI SCIENCES, EK210).

**Statistical analysis**

GraphPad Prism 10 software was utilized for statistical analysis. All data are presented as mean ± Standard Error of the Mean (SEM). One-way analysis of variance (ANOVA) analysis followed by Tukey’s post-hoc test were employed. Analysis items with *P* < 0.05 were considered statistically significant.

References

1 Qing, H. et al. Valproic acid inhibits Abeta production, neuritic plaque formation, and behavioral deficits in Alzheimer's disease mouse models. *J Exp Med* **205**, 2781-2789 (2008).

2 Sun, X. et al. Hypoxia facilitates Alzheimer's disease pathogenesis by up-regulating BACE1 gene expression. *Proc Natl Acad Sci U S A* **103**, 18727-18732 (2006).

3 Ly, P. T. et al. Inhibition of GSK3beta-mediated BACE1 expression reduces Alzheimer-associated phenotypes. *J Clin Invest* **123**, 224-235 (2013).

4 Zhang, Y. et al. Activation of AMPK by GLP-1R agonists mitigates Alzheimer-related phenotypes in transgenic mice. *Nat Aging* **5**, 1097-1113 (2025).

5 Xia, L. et al. Disruption of BAG3-mediated BACE1 stabilization alleviates neuropathology and memory deficits in a mouse model of Alzheimer's disease. *Sci Adv* **11**, eadt7981 (2025).

6 Feng, Y. et al. Inhibition of IFITM3 in cerebrovascular endothelium alleviates Alzheimer's-related phenotypes. *Alzheimers Dement* **21**, e14543 (2025).

7 Luo, M. et al. miR-429-3p mediates memory decline by targeting MKP-1 to reduce surface GluA1-containing AMPA receptors in a mouse model of Alzheimer's disease. *Acta Pharm Sin B* **14**, 635-652 (2024).
